# Fluorescence lifetime imaging of NAD(P)H upon oxidative stress in *Kluyveromyces marxianus*

**DOI:** 10.3389/fbioe.2022.998800

**Published:** 2022-09-02

**Authors:** Yi Ai, Ruoyu Luo, Deqiang Yang, Jiong Ma, Yao Yu, Hong Lu

**Affiliations:** ^1^ State Key Laboratory of Genetic Engineering, School of Life Sciences, Fudan University, Shanghai, China; ^2^ Shanghai Engineering Research Center of Industrial Microorganisms, Fudan University, Shanghai, China; ^3^ Key Laboratory of Micro and Nano Photonic Structures (Ministry of Education), Shanghai Engineering Research Center of Ultra-precision Optical Manufacturing, Department of Optical Science and Engineering, School of Information Science and Technology, Fudan University, Shanghai, China

**Keywords:** *Kluyveromyces marxianus*, oxidative stress, NAD(P)H autofluorescence, FLIM, heterologous protein

## Abstract

*K. marxianus* is a promising cell factory for producing heterologous proteins. Oxidative stresses were raised during overexpression of heterologous proteins, leading to the shift of the redox state. How to measure the redox state of live *K. marxianus* cells without perturbing their growth remains a big challenge. Here, a fluorescence lifetime imaging (FLIM)-based method was developed in live *K. marxianus* cells. During the early exponential growth, *K. marxianus* cells exhibited an increased mean fluorescence lifetime (τ-mean) of NAD(P)H compared with *Saccharomyces cerevisiae* cells, which was consistent with the preference for respiration in *K. marxianus* cells and that for fermentation in *S. cerevisiae* cells. Upon oxidative stresses induced by high temperature or H_2_O_2_, *K. marxianus* cells exhibited an increased τ-mean in company with decreased intracellular NAD(P)H/NAD(P)^+^, suggesting a correlation between an increased τ-mean and a more oxidized redox state. The relationship between τ-mean and the expression level of a heterologous protein was investigated. There was no difference between the τ-means of *K. marxianus* strains which were not producing a heterologous protein. The τ-mean of a strain yielding a high level of a heterologous protein was higher than that of a low-yielding strain. The results suggested the potential application of FLIM in the non-invasive screen of high-yielding cells.

## Introduction

*K. marxianus* is a promising microbial cell factory for producing heterologous proteins, bioethanol and bulk chemicals (Karim et al., 2020; Leonel et al., 2021). During the production of heterologous proteins, yeast faces various extracellular disturbances, including high temperature, high salt and abnormal pH, which imbalance the redox state of yeast cells (Qiu et al., 2019). For example, high temperature increases the production of intracellular reactive oxygen species (ROS), causing oxidative stress to the yeast (Fu et al., 2019). A high concentration of salt disturbs redox homeostasis, leading to oxidative damage (Ramos-Moreno et al., 2019). Meanwhile, oxidative stresses are invoked endogenously during the overexpression of heterologous proteins, because the increased demand for NAD(P)H is raised in different steps of protein production, including amino acid biosynthesis (Zhu et al., 2021), disulfide bond formation (Miller et al., 2018), and protein secretion (Tomàs-Gamisans et al., 2020). Therefore, measuring the redox state of live *K. marxianus* cells might be applied to monitor the production of heterologous proteins upon various disturbances.

Chemical and optical methods have been developed to assess the redox state of cells. Chemical methods indirectly infer the redox state of the cell mainly by measuring the concentration of redox pairs such as NAD(P)H/NAD(P)^+^ (Wang et al., 2017). Optical methods include fluorescent biosensors and autofluorescence methods. Fluorescent biosensors assess redox status at the single-cell level by transferring fluorescent protein-encoding genes (Liao et al., 2020). Using NAD(P)H autofluorescence to assess cellular redox status emerges as a promising method in recent years, as it enables endogenous noninvasive indication of the redox status. NAD(P)H displays a maximum autofluorescence emission wavelength at 450–535 nm when using the single-photon excitation at 340 nm (Cannon et al., 2021). Fluorescence lifetime imaging (FLIM) measures the mean fluorescence lifetime (τ-mean) of NAD(P)H. The lifetime (τ) of free NAD(P)H (τ-free) is usually fixed at about 0.4 ns The τ of bound NAD(P)H (τ-bound) varies with different combined proteins and is usually around 1–4 ns (Schaefer et al., 2019). The ratio of free NAD(P)H and bound NAD(P)H to the total intracellular NAD(P)H is defined as a1 and a2, respectively (Kolenc and Quinn, 2019). a1/a2 is correlated with NAD(P)H/NAD(P)^+^, which defines the redox state of cells (Sant’Anna-Silva et al., 2018). Usually, a more oxidized state increases both τ-mean and τ-bound, while reducing a1/a2 (Schaefer et al., 2017; Meleshina et al., 2018). However, there are few reports about the FILM analysis in living yeast cells, including *K. marxianus* cells (Kong et al., 2022).

In this study, the redox state of *K. marxianus* cells was assessed by FLIM of NAD(P)H. *K. marxianus* cells exhibited an increased τ-mean of NAD(P)H compared with *S. cerevisiae* cells during early exponential growth, which was consistent with different Crabtree-effect statuses of two yeasts. *K. marxianus* cells exhibited an increased τ-mean upon oxidative stresses induced by high temperature or H_2_O_2_, suggesting a correlation between an increased τ-mean and a more oxidized redox state. Notably, an increased τ-mean was correlated the high-level expression of a heterologous protein in *K. marxianus* cells, which suggested a potential application of FLIM in the non-invasive screen of high-yielding cells.

## Materials and methods

### Yeast strains and plasmids

The *K. marxianus* strain FIM-1 (Wu et al., 2020), and the *S. cerevisiae* strain S288C (Yu et al., 2021), were used in this study. *K. marxianus* strains KML and KMH were derived from KM-HPV2, which expressed IBDV-VP2 by pUKDN115 (Yang et al., 2021). Plasmid expressing IBDV-VP2 was replaced by the void pUKDN115 in KML and KMH to obtain KML_void_ and KMH_void_, respectively.

### Medium and culturing

To compare the redox states of *K. marxianus* and *S. cerevisiae* cells, FIM-1 and S288C cells were grown in a YPD medium (20 g/L tryptone, 10 g/L yeast extract, 20 g/L glucose) at 30°C overnight and the culture was diluted into a fresh YPD medium to start at an OD_600_ of 0.1. Cells were then grown at 30°C for 3 h. To compare redox states at different temperatures, overnight culture of FIM-1 was inoculated into a fresh YPD medium to start at an OD_600_ of 0.1 and then grown at 30°C or 45°C for 24 h. To perform the H_2_O_2_ treatment, FIM-1 cells were grown at 30°C for 24 h and then grown in the presence of 20 mM H_2_O_2_ for 1 h. Untreated culture served as a control. To compare redox states during expressing IBDV-VP2, KML and KMH cells were grown in the YD medium (20 g/L yeast extract, 40 g/L glucose) for 3 days. Cells were collected after 1 or 3 days.

### Measurement of dissolved oxygen, residual glucose and ethanol

The culture of FIM-1 and S288C was centrifuged and the supernatant was subjected to analysis. The amount of dissolved oxygen was measured by a dissolved oxygen analyzer (JPSJ-605F, Shanghai INESA Scientific Instrument, China) according to the manufacturer’s manual. Residual glucose and ethanol was measured on high-performance liquid chromatography (1260, Agilent, United States) as described before (Ai et al., 2022).

### Preparation of yeast cells for fluorescence lifetime imaging

To immobilize yeast cells, 20–25 μl of ConA (10 mg/ml, C2010, Sigma-Aldrich, United States) was spread onto the bottom of a glass-bottom petri dish (801001, NEST, China) and the dish was left to dry overnight at room temperature. Yeast cells were pelleted and resuspended in sterile water to an OD_600_ of 5. A total of 5 μl sample was transferred to the ConA-coated dish for analysis.

### Fluorescence lifetime imaging imaging and data analysis

FLIM was performed by a Leica TCS SP8 DIVE FALCON microscope (Leica, Germany) equipped with a ×63 water immersion objective (N.A. = 1.2) excited by a femtosecond pulsed laser with ∼3 mW power at 740 nm (InSight X3 Dual, Spectra-Physics, United States). The FLIM images were exported in lif format and ROIs were selected for processing in Leica Application Suite X software to obtain fluorescence lifetime data. The sizes of experimental ROIs samples ranged from 52 to 104. The data were fitted using a double exponential model as τ-mean = a1 τ-free + a2 τ-bound (Kong et al., 2022). Statistical analyses were performed by GraphPad-Prism version 8.0 software (GraphPad, United States). As to the significance test, data that did not follow a normal distribution was tested using the nonparametric Mann-Whitney test. Data that followed a normal distribution and satisfied the homogeneity of variances was tested using an unpaired *t*-test. Otherwise, Welch’s modified unpaired *t*-test was used.

### Measurement of intracellular concentrations of NAD(P)^+^ and NAD(P)H

Concentrations of intracellular NAD^+^, NADH, NADP^+^ and NADPH were determined by a WST-8 based colorimetric assay using commercial kits (S0175 and S0179, Beyotimes, China). To collect cells grown at different temperatures and cells treated with H_2_O_2_, FIM-1 cells were cultured as described above in “Medium and culturing”. Cells of 20 OD_600_ were collected, washed and then suspended in 200 μl extraction buffer provided by the kit. Cells were then mixed with 200 μl acid-washed glass beads (G8772, Sigma-Aldrich, United States) and processed by a bead-beater (FastPrep-24, MP, United States) at 6 m/s for 2 min. The lysate was centrifuged at 13,200 rpm for 10 min at 4°C. A total of 20 and 50 μl supernatant was collected for NAD^+^/NADH and NADP^+^/NADPH assay, respectively. Experiments were performed in triplicates and repeated three times. Ratios of NADH/NAD^+^, NADPH/NADP^+^ and NAD(P)H/NAD(P)^+^ were calculated based on the concentrations.

## Results

### The redox states of *K. Marxianus* and *S. cerevisiae* cells could be differentiated by the τ-mean of NAD(P)H

**FIGURE 1:**
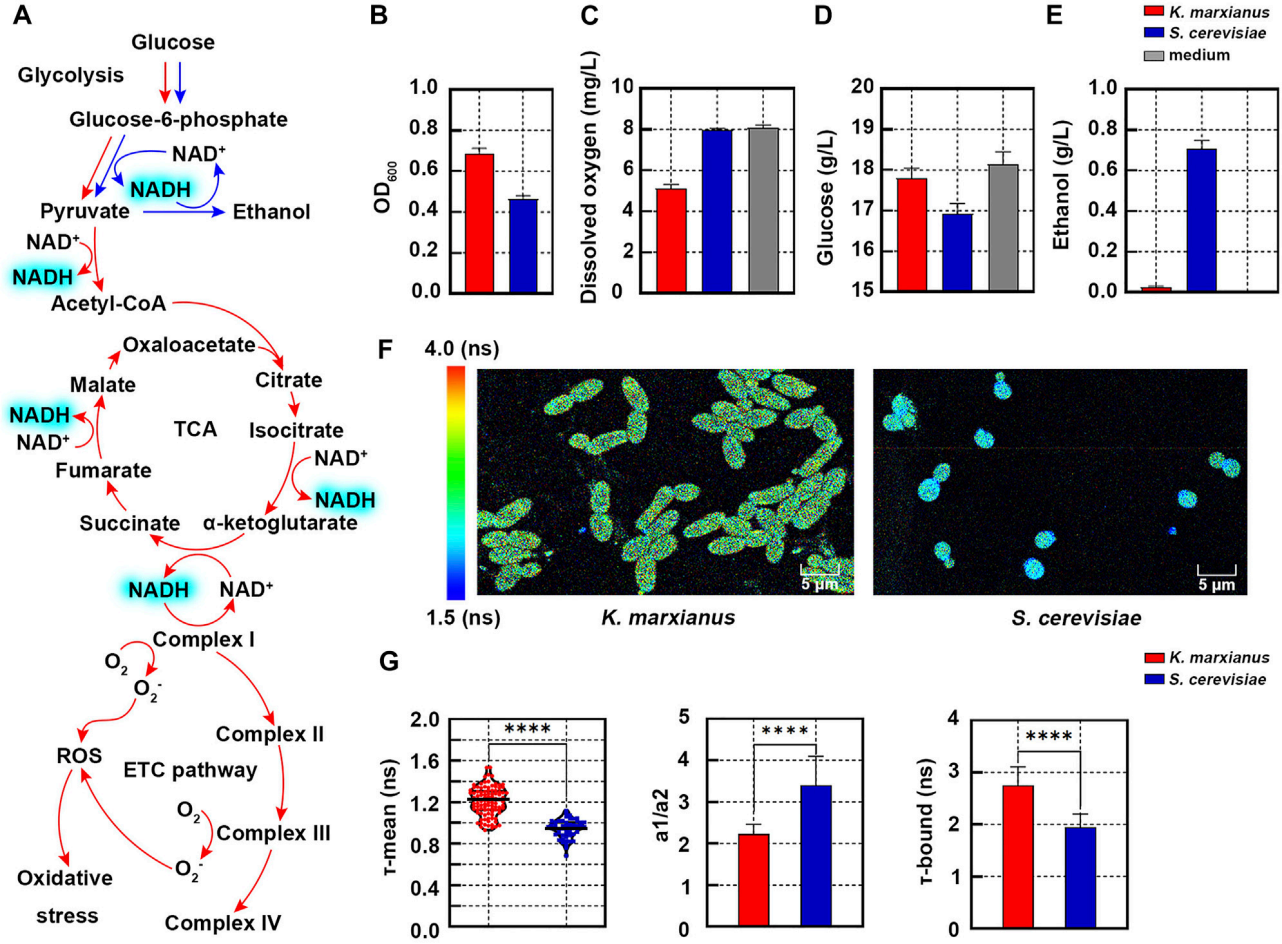
Redox states of *K. marxianus* and *S. cerevisiae* cells characterized by FLIM. **(A)** Aerobic respiration (red) and ethanol fermentation (blue) pathways in yeast. **(B–E)** OD_600_ **(B)**, dissolved oxygen **(C)**, residual glucose **(D)** and ethanol **(E)** in the culture of *K. marxianus* (FIM-1) and *S. cerevisiae* (S288C) cells after 3 h of growth. Medium without inoculation served as a control. Values represented mean ± SD (*n* = 3). **(F)** FLIM of NAD(P)H in *K. marxianus* and *S. cerevisiae* cells after 3 h of growth. Pseudocolor mapping of NAD(P)H fluorescence lifetimes was shown. The lifetime of NAD(P)H from 1.5 to 4.0 ns was assigned different colors. **(G)** Quantitative analysis of NAD(P)H fluorescence in cells from **(F)**. A violin plot showed the distribution of τ-mean of cells and the mean value was indicated by a black line. Histograms showed a1/a2 and τ-bound, in which values represented mean ± SD. A total of 101 *K. marxianus* cells and 52 *S. cerevisiae* cells were counted. ****p < 0.0001.

In yeast, the metabolic flow of glucose between ethanol fermentation and aerobic respiration influences the redox state of cells. In ethanol fermentation, 1 molecule of glucose produces 2 molecules of NADH through glycolysis and NADH is subsequently oxidized by ethanol dehydrogenase to regenerate NAD^+^ (Figure 1A). In comparison, 10 molecules of NADH are produced in aerobic respiration and oxidized by oxidative phosphorylation, which increases the chance to produce reactive oxygen species (ROS) and biases the cell toward a more oxidized state (Scialò et al., 2017) (Figure 1A).

Metabolic flows of glucose in *S. cerevisiae* cells (SC228C) and *K. marxianus* cells (FIM-1) during the early exponential growth were investigated in the first place. After 3 h of growth in the YPD medium (containing 20 g/L glucose), the OD_600_ of *K. marxianus* cells was 1.49 times that of *S. cerevisiae* cells (Figure 1B). Compared with the medium without inoculation, dissolved oxygen in the culture of *S. cerevisiae* cells decreased by 1.36%, while that of *K. marxianus* cells decreased by 36.5% (Figure 1C). The amount of glucose consumed by *K. marxianus* cells was 29% of that by *S. cerevisiae* cells (Figure 1D). The concentration of ethanol in the culture of *S. cerevisiae* cells was 0.71 g/L, while there was hardly any ethanol produced by *K. marxianus* cells (Figure 1E). The results suggested that *S. cerevisiae* cells preferred fermentation in the aerobic condition with excess glucose, which was consistent with the Crabtree-positive identity of *S. cerevisiae* (Dai et al., 2018). Meanwhile, results suggested that *K. marxianus* cells preferentially used respiration in the presence of oxygen and glucose, which was consistent with the Crabtree-negative identity of *K. marxianus* (Sakihama et al., 2019; Yu et al., 2021). Different ways to assimilate glucose suggested distinct redox states in *S. cerevisiae* and *K. marxianus* cells.

To compare the redox states of two yeasts, the fluorescence lifetime of NAD(P)H in cells was analyzed by FLIM. In the pseudocolor mapping of NAD(P)H lifetime, *K. marxianus* cells displayed yellow-green and *S. cerevisiae* cells displayed light blue (Figure 1F), suggesting NAD(P)H lifetime of *K. marxianus* cells was longer. Consistently, τ-means of NAD(P)H *in K. marxianus and S. cerevisiae* cells were quantified as 1.21 ± 0.27 ns and 0.94 ± 0.19 ns, respectively (Figure 1G, left). The a1/a2 of *K. marxianus* cells (2.23) was significantly lower than that in *S. cerevisiae* cells (3.40) (Figure 1G, middle), suggesting the redox state of *K. marxianus* cells was relatively more oxidized. The lower a1/a2 of *K. marxianus* cells was mainly due to significantly increased τ-bound of NAD(P)H (Figure 1G, right), as more NAD(P)H might bind with enzymes during aerobic respiration in *K. marxianus* cells. In general, the redox state of *K. marxianus* and *S. cerevisiae* cells could be differentiated by the τ-mean of NAD(P)H, as a longer τ-mean of *K. marxianus cells* suggested a more oxidized state.

### *K. marxianus* cells exhibited an increased τ-mean of NAD(P)H upon oxidative stress induced by high temperature or H_2_O_2_ treatment

**FIGURE 2:**
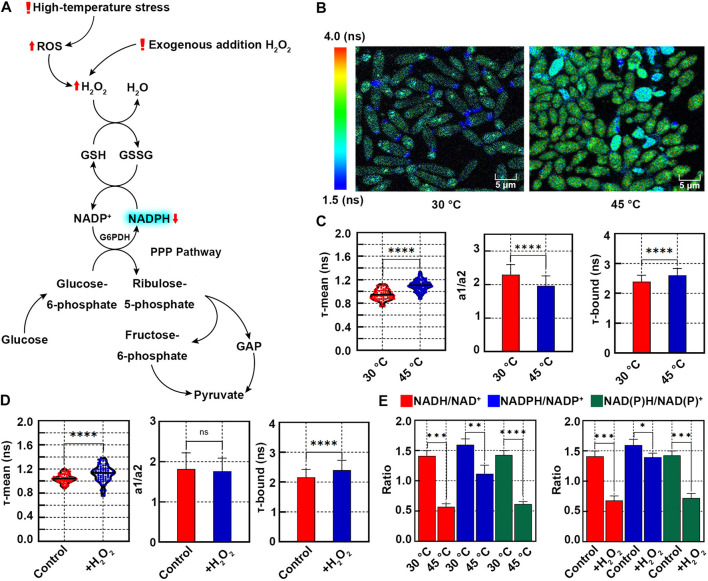
Redox states of *K. marxianus* cells upon oxidative stress induced by high-temperature or H_2_O_2_ treatment. **(A)** Oxidative stress induced by high temperature or H_2_O_2_ leads to the increased consumption of NADPH. **(B)** FLIM of NAD(P)H in cells grown at 30°C or 45°C. Pseudocolor mapping of NAD(P)H fluorescence lifetimes was shown. The lifetime from 1.5 to 4.0 ns was assigned different colors. **(C)** Quantitative analysis of NAD(P)H fluorescence lifetimes of cells in **(B)**. A violin plot showed the distribution of τ-mean of cells and the mean value was indicated by a black line. Histograms showed a1/a2 and τ-bound, in which values represented mean ± SD. A total of 68 cells grown at 30°C and 112 cells grown at 45°C were counted. ****p < 0.0001. **(D)** Quantitative analysis of NAD(P)H fluorescence lifetimes of cells treated with H_2_O_2_. A total of 66 control cells and 69 cells treated with H_2_O_2_ were counted. “ns” indicated *p* > 0.05. **(E)** Ratios of NADH/NAD^+^, NADPH/NADP^+^ and NAD(P)H/NAD(P)^+^ of cells grown at high temperature or treated with H_2_O_2_. Concentrations of intracellular NAD^+^, NADH, NADP^+^ and NADPH were measured by a chemical assay. Ratios were calculated based on the concentrations. Values represented mean ± SD (*n* = 3). *p < 0.05; **p < 0.01; ***p < 0.001.

High temperature and H_2_O_2_ induced oxidative stress in *K. marxianus* (Arellano-Plaza et al., 2017; Fu et al., 2019). Increased consumption of free NADPH is required to alleviate oxidative stress (Figure 2A), which is expected to shift the redox state (Mara et al., 2018).

To investigate the redox state at a high temperature, *K. marxianus* cells (FIM-1) were grown at 30°C or 45°C for 24 h. Pseudocolor mapping of NAD(P)H lifetime of cells at 30°C showed dark blue and faint cyan, indicating a shorter lifetime (Figure 2B). Most of the cells at 45°C displayed yellow-green, indicating a longer lifetime (Figure 2B). The τ-mean of NAD(P)H of cells grown at 30°C was 0.94 ± 0.09 ns and that of cells grown at 45°C was significantly increased to 1.10 ± 0.09 ns (Figure 2C, left), suggesting a relative oxidative state of cells at high temperature. The a1/a2 of cells grown at 45°C was significantly lower than that at 30°C (Figure 2C, middle). τ-bound of cells grown at 45°C was significantly longer than that at 30°C (Figure 2C, right).

Similar to the situation of cells grown at a high temperature, the τ-mean of NAD(P)H of cells treated with 20 mM H_2_O_2_ (1.12 ± 0.13 ns) was significantly longer than that in untreated cells (1.04 ± 0.07 ns) (Figure 2D, left). τ-bound of treated cells was significantly longer than that of control cells (Figure 2D, right). However, a1/a2 of treated cells did not change significantly (Figure 2D, middle), probably due to the increased fluctuation of cell statuses upon H_2_O_2_ treatment.

To confirm the relationship between NAD(P)H content and τ-mean, concentrations of intracellular NADH, NAD^+^, NADPH and NADP^+^ were determined by a colorimetric chemical assay. As shown in Figure 2E, NADH/NAD^+^, NADPH/NADP^+^ and NAD(P)H/NAD(P)^+^ of cells grown at 45°C were all significantly decreased, compared with their counterparts of cells grown at 30°C. Similarly, H_2_O_2_ treatment significantly decreased NADH/NAD^+^, NADPH/NADP^+^ and NAD(P)H/NAD(P)^+^ of cells (Figure 2E, right). The results were consistent with increased consumption of free NADPH upon oxidative stress induced by high temperature or H_2_O_2_ (Figure 2A). Therefore, an increased τ-mean of NAD(P)H of *K. marxianus* cells could be applied to indicate a shift in redox state [defined by NAD(P)H/NAD(P)^+^] toward a more oxidative status.

### *K. marxianus* cells with a high yield of a heterologous protein exhibited an increased τ-mean of NAD(P)H

Overexpressing heterologous proteins raised oxidative stress in yeast (Huang et al., 2017). To compare redox states of cells displaying different expression levels of a heterologous protein, two *K. marxianus* strains, KML and KMH were selected. In our previous study, KM-HPV2 was constructed to express infectious bursal disease virus (IBDV) capsid VP2 by an episomal vector (Yang et al., 2021). KM-HPV2 was subjected to the H_2_O_2_ mutagenesis. Among the mutated strains, KML and KMH displayed low and high-level expressions of IBDV-VP2, respectively. As controls, VP2-expressing vectors in KML and KMH strains were replaced by void vectors to obtain KML_void_ and KMH_void_, respectively.

**FIGURE 3:**
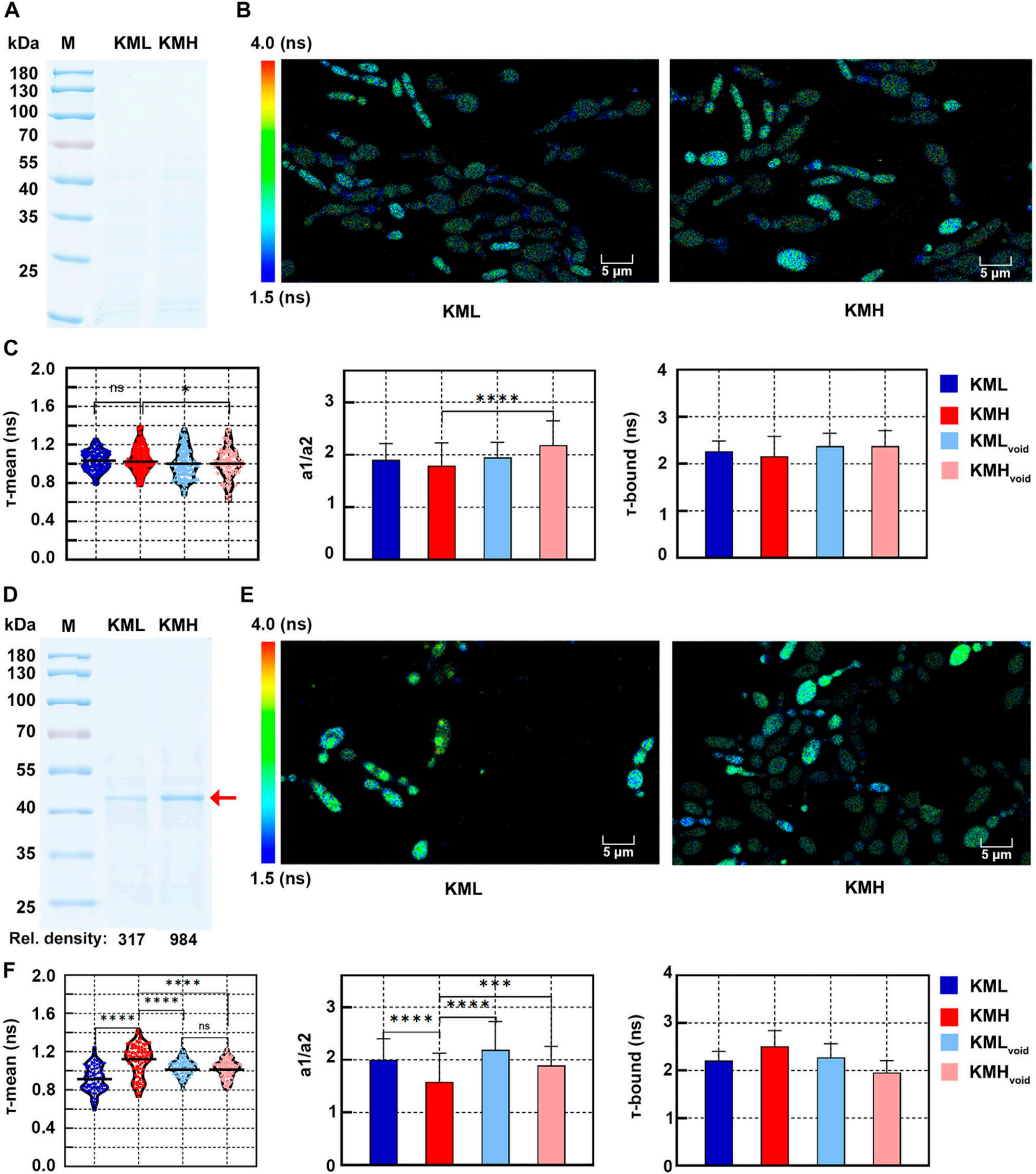
Redox states of *K. marxianus* cells displaying different expression levels of a heterologous protein. **(A)** SDS-PAGE of supernatant of KML and KMH culture after 1 day of growth. **(B)** FLIM of NAD(P)H in KML and KMH cells after 1 day of growth. Pseudocolor mapping of NAD(P)H fluorescence lifetimes was shown. The lifetime from 1.5 to 4.0 ns was assigned different colors. **(C)** Quantitative analysis of NAD(P)H fluorescence lifetimes of cells after 1 day of growth. A violin plot showed the distribution of τ-mean of cells and the mean value was indicated by a black line. Histograms showed a1/a2 and τ-bound, in which values represented mean ± SD. A total of 94 KML, 105 KMH, 104 KML_void_ and 78 KMH_void_ cells were counted. “ns” indicated *p* > 0.05. *p < 0.05; ****p < 0.0001. **(D)** SDS-PAGE of supernatant of KML and KMH culture after 3 days of growth. The position of IBDV-VP2 was indicated by a red arrow and the relative densities of bands were shown below. **(E)** Pseudocolor mapping of NAD(P)H fluorescence lifetimes of KML and KMH cells after 3 days of growth. **(F)** Quantitative analysis of NAD(P)H fluorescence lifetimes of cells after 1 day of growth. A total of 91 KML, 79 KMH, 96 KML_void_ and 102 KML_void_ cells were counted. ***p < 0.001.

After 1 day of growth, IBDV-VP2 could not be detected in the supernatant of KML or KMH culture in SDS-PAGE (Figure 3A), suggesting the expression of IBDV-VP2 in either strain was low. In the pseudocolor mapping of NAD(P)H lifetime, most KML and KMH cells displayed blue-green, indicating a short lifetime (Figure 3B). In the quantitative analysis (Figure 3C), there was no significant difference between the τ-mean of KMH (1.05 ± 0.13 ns) cells and that of KML (1.03 ± 0.10 ns) cells, suggesting both cells exhibited similar redox states while expressing low levels of IBDV-VP2. However, the τ-mean of KMH cells was significantly longer than that of KMH_void_ cells (1.00 ± 0.16 ns) (Figure 3C, left). Consistently, a1/a2 of KMH cells was significantly lower than that of KMH_void_ cells (Figure 3C, middle). It suggested the redox state of KMH cells was more oxidized than that of KMH_void_ cells, which might be related to IBDV-VP2 produced in KMH cells.

After 3 days of growth, bands corresponding to IBDV-VP2 were visible in the supernatant of KML and KMH culture in SDS-PAGE. The amount of IBDV-VP2 produced by KMH cells was 3 times that by KML cells, as deduced by the relative densities of bands (Figure 3D). A fraction of KML and KMH cells displayed yellow-green in the pseudocolor mapping of NAD(P)H lifetime (Figure 3E), suggesting a longer lifetime compared with that of cells after 1 day of growth. In the quantitative analysis (Figure 3F, left), the τ-mean of KMH cells (1.11 ± 0.17 ns) was significantly higher than that of KML (0.91 ± 0.13 ns), KMH_void_ (1.02 ± 0.09 ns) and KML_void_ cells (1.03 ± 0.08 ns). The distribution of τ-mean of KMH_void_ and KML_void_ cells was more concentrated. It suggested that, after entering the stationary phase, cells without expressing a heterologous protein exhibited a redox state with less fluctuation. Consistent with the highest τ-mean of KMH cells, a1/a2 of KMH cells was the lowest and τ-bound of KMH cells was the longest among their counterparts (Figure 3F, middle and right). The results suggested that, compared with cells with low or no expression of a heterologous protein, KMH cells with a high yield of a heterologous protein exhibited a more oxidized state, which could be indicated by an increased τ-mean of NAD(P)H.

## Discussion

Since Britton Chance discovered the autofluorescence of NAD(P)H in the 1950s (Chance, 1952), methods to analyze the cellular redox state analysis based on NAD(P)H autofluorescence, such as FLIM, have been gradually developed (Tornmalm et al., 2019). FLIM is a non-invasive, label-free and *in situ* method, which is suitable for the long-term detection of living cells and therefore is widely applied in clinical research. For example, tumor cells tend to undergo glycolysis, the so-called Warburg effect, which leads to a low fluorescence lifetime of NAD(P)H (Kolenc and Quinn, 2019). Therefore, FLIM is used for early tumor detection (Alfonso-García et al., 2022). The redox states of stem cells change considerably during differentiation and FLIM was applied to track the metabolism changes of stem cells (Okkelman et al., 2020). Yeast is one of the most important microbial cell factories and the yielding of yeast cells was closely related to redox states (Chen et al., 2020). FLIM is well suited to measure the redox state of yeast, which might benefit strain engineering for improved yield. However, few applications of FLIM in yeast were reported so far.

In this study, FLIM was performed to assess the redox states of *K. marxianus* cells upon various oxidative stress. Among the current methods to analyze the redox states of cells, chemical analysis destroys cell integrity and does not allow for single-cell analysis (Kanamori et al., 2018). Although fluorescent biosensors can assess redox states at the single-cell level, they require a long preparation procedure and the introduction of exogenous genes (Liao et al., 2020). In comparison, FILM gives a more natural image of the cell state. For example, high-temperature stress resulted in some cells turning blue in the FLIM (Figure 2B), implying the presence of a large amount of short-lived NAD(P)H in these cells even τ-mean of NAD(P)H of all cells increased. Both high-temperature and H_2_O_2_ treatment led to an increase of τ-mean of NAD(P)H. But H_2_O_2_ caused more deviation of τ-mean than high temperature, suggesting more fluctuated redox states were raised among the cells treated by H_2_O_2_ (Figures 2C,D). There were also shortcomings of FLIM in assessing the redox states of cells. For example, the autofluorescence of NADH and NADPH is optically indistinguishable and therefore FLIM can not analyze individual redox pairs directly. In addition, the fluctuation of τ-mean among individual cells was considerable. Some fluctuation might be due to the genetic diversity among cells, as observed in the offspring of one mother cell (Maclean et al., 2017). Meanwhile, some fluctuation might be due to different phases in the cell cycle, different ages of cells, various epigenetic statuses and some technical issues, such as errors in detecting photorefractive rate. Therefore, synchronization of cells and a more accurate detection apparatus might help to reduce the fluctuation of τ-mean among the population.

Overproduction of heterologous proteins resulted in the reduction of NADPH and that led to an altered redox cofactor state (Tomàs-Gamisans et al., 2020). Consistent with this idea, *K. marxianus* cells with a high yield of a heterologous protein exhibited an increased τ-mean of NAD(P)H (Figure 3F). Therefore, τ-mean of NAD(P)H might be used as an intrinsic marker to screen high-yielding cells. Traditionally, cells expressing heterologous proteins are verified by SDS-PAGE, which was laborious and not suitable for high-throughput screens. High-throughput screen techniques, such as microfluidics, were developing rapidly in recent years, in which fluorescent markers are usually required (Napiorkowska et al., 2021). For example, fluorescent groups released after enzymatic reactions are applied to the screen of enzymes. But this strategy is not applicable for the screen of non-enzymatic proteins, including antibodies, cytokines and vaccines. Fluorescent fusion proteins, such as GFP-fused proteins, are compatible with the high-throughput screen (Baumann et al., 2018). However, fluorescent fusion proteins sometimes inhibit the natural activity of target proteins (Wick et al., 2019). Since FLIM analysis of NAD(P)H does not require the fluorescent labelling of target proteins or their products, it might be applied to the screen of cells yielding the target protein in its natural form. With the aid of machine learning algorithms and robotic platforms, automatic FLIM analysis and screen are possible (Sagar et al., 2020). If a more general relationship between high-yielding cells and an increased τ-mean of NAD(P)H is confirmed in the future, a FLIM-based screen will boost the optimization of yeast cell factories.

## Data Availability

The original contributions presented in the study are included in the article/Supplementary Material, further inquiries can be directed to the corresponding authors.
